# Organization of the cancer network in SUS: evolution of the care model

**DOI:** 10.6061/clinics/2018/e430s

**Published:** 2018-09-17

**Authors:** Marisa Riscalla Madi, Giovanni Guido Cerri

**Affiliations:** I Instituto de Radiologia InRad, Hospital das Clinicas HCFMUSP, Faculdade de Medicina, Universidade de Sao Paulo, Sao Paulo, SP, BR; II Departamento de Radiologia, Faculdade de Medicina FMUSP, Universidade de Sao Paulo, Sao Paulo, SP, BR

**Keywords:** Medical Oncology, Health Services, Health Policy, Integrated Health Care

## Abstract

In the current context of epidemiological transition, demographic changes, changes in consumption and lifestyle habits, and pressure on care costs and organized health systems for acute conditions, the Integrated Care Model by Shortell has become a conceptual reference in the search for new methods to manage chronic conditions by focusing on the health conditions of a given population that must be addressed by a set of institutions organized into networks. Within the last 15 years, cancer has gone from the third- to the second-leading cause of death in the State of São Paulo and has shown a gradual increase in the number of new cases; it has thus become a relevant issue for public health and health management. The model adopted by the State for the organization of the cancer care network was the motivation for this study, which aimed to evaluate the evolution of the model of care for cancer patients within the Unified Health System (Sistema Único de Saúde) based on the integrated care model. Since 1993, the year that cancer was first considered highly complex in the Sistema Único de Saúde by the Ministry of Health, it has been possible to observe a progressive orientation towards the integral and integrated care of patients with cancer. In the State of São Paulo, the active participation of qualified service providers through a Technical Reference Committee showed that experts could contribute to the definition of public policies, thereby providing a technical base for decision making and contributing to the development of clinical management.

## INTRODUCTION

In the current context of epidemiological transition coupled with demographic changes (aging of the population and urbanization), changes in consumption and lifestyle habits, increasing pressure on care costs, a more rights-conscious population, and organized health systems, the World Health Organization (WHO) has proposed the Model of Innovative Care for Chronic Conditions, focusing on population, promotion, prevention and the integration of care 1.

**Figure 1:**
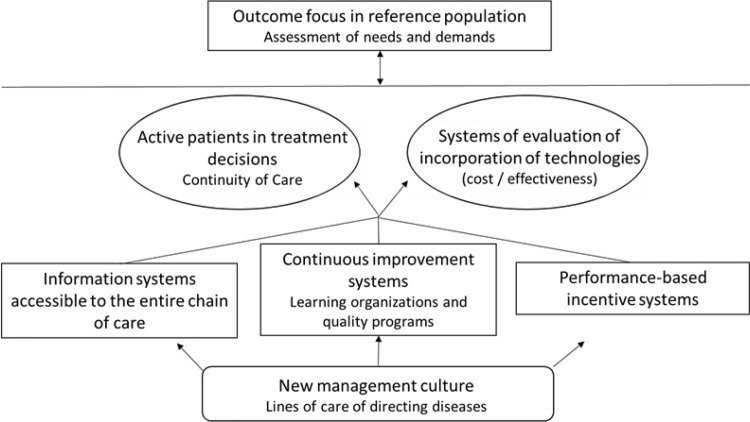
The seven elements of Shortell for integrating health care. Source: Prepared by the authors based on Shortell, 1993 2.

According to the model developed by Shortell, there are seven elements of the integration of care, as shown in Figure 1. Thus, institutions should be organized into networks to ensure a “coordinated continuum of services” 2.

The challenge is the transformation of an acute case-focused system with an emphasis on disease and individual planned care whose primary goal is to fill hospital beds and whose organization is departmentalized and focused on the functioning of institutions in isolation. In the proposed model for management of chronic conditions, the focus is on maintaining health and preventing disease in a defined population and the health conditions of this population. A set of institutions has been organized into networks to manage this model.

Among the barriers to integration are the inability to understand the new business model in which primary care is the center of care and the well-being of the target population becomes the focus of the expected results. Thus, there is a need for all health professionals to act in an integrated manner. Hospitals must stop acting alone and must establish alliances with other hospitals and clinics in the community while developing a specific strategy for primary care. The greatest barrier in the US Academic Health Systems is cultural. These systems are characterized by the strong fragmentation of care and a high level of knowledge in inpatient treatment with high complexity (with high use of technology). In addition, the new demands require knowledge to act in the low-complexity (and low-technology) situations of primary care 3.

In Brazil, since the conception of the Unified Health System (Sistema Unico de Saúde; SUS), the Healthcare System of Brazil, it has been organized based on regions and networks. In the English model of Regionalised and Hierarchical Networks, there is a network of regions based on large territories with primary health centers, secondary schools and teaching hospitals that ensure access to comprehensive care and seek self-sufficiency in health resources at all levels and in smaller territorial subdivisions. Recently, under the influence of the Shortell model, the concept by Mendes has become the basis for the guidelines of the SUS organization in Health Care Networks 4.

In the Pact for Health in 2006, a regulatory framework was established that encouraged the creation of models capable of responding to chronic and acute conditions and that promote health surveillance and actions 5. In 2010, a Ministerial Decree defined the Health Care Networks 6, and in 2011, the decree that regulated Law 8080, the SUS Law, defined the Health Regions and Health Care Networks 7. This legal set aims to reduce the inequalities imposed by the territory where the individual lives, ensuring citizens' access to necessary actions and health services close to where they live 8.

In the State of São Paulo, based on this legal framework, 17 Regional Health Care Networks (Redes Regionais de Aatenção à Saúde; RRAS) were established from a grouping of 63 Health Regions after an extensive process of regional discussion and agreement 9,10.

Each RRAS consists of health services of different technological densities and of support systems to ensure the integrality of the services. Established organized, systematized and regulated horizontal relations between basic care and these different health services are recognized as points of attention. Each RRAS must have a sufficient capacity for basic care, medium-complexity services and some high-complexity services. It is also within these territories that Thematic Networks must be organized (e.g., Urgency and Emergency, Maternal and Child, and Oncology), some of which are restricted to high-complexity services and others include services of different levels of complexity.

In the past 15 years, cancer has gone from the third- to second-leading cause of death in the state and has shown a gradual increase in the number of new cases. For this reason, the study of the Thematic Oncology Network is relevant for public health and health management. This study aims to evaluate the evolution of the care model for cancer patients in SUS based on the integrated care model.

## METHODOLOGY

For the theoretical basis, the topic of health care networks and oncological assistance was studied via a review of the literature in the databases of Bireme - Virtual Health Library (VHL) and PubMed, a survey of the Brazilian standards for care (Laws, Decrees, Ordinances and Resolutions), reports of the WHO and publications and reports of the National Cancer Institute (Instituto Nacional do Câncer; INCA).

## RESULTS

In Brazil, cancer ceased to be a disease treated only in medical offices and became a public health problem in 1922 with the Anticancer First Plan and the inauguration of the Radium Institute in Belo Horizonte, a private entity and the first center to fight cancer in the country. In 1941, the National Cancer Service (SNC) was created to organize, guide and control the anticancer campaign at the national level. The SNC obtained its headquarters in 1957 with the inauguration of the INCA to direct national policy for the control of cancer from 1990 with the promulgation of the Organic Health Law 11. From the perspective of health services and systems planning, the cancer care line involves all levels in different services and stages, with multiprofessional involvement and multiple medical specialties, in a non-linear process.

The complexity involved in the different stages is a risk factor in terms of time, a prognostic element in regard to cancer. The structures necessary for the treatment of cancer patients are highly complex. The modalities can vary between surgery, radiotherapy and/or chemotherapy in different combinations according to each case. For the provision of cancer care under the SUS, there is a need for a specific authorization determined by technical criteria presented in the Ministerial Orders. From this qualification, hospitals receive authorization to bill the procedures performed according to the SUS Procedure Table using the Authorisation for High Complexity Procedures in Oncology (Autorização de Procedimento de Alta Complexidade; APAC Oncology) 12,13.

Since 1993, when the Ministry of Health classified Oncology as an area of high complexity, a series of regulatory instruments have been published to direct service providers and system managers with regard to the organization of patient care. Table 1 summarizes each of these publications 14-19.

The last Ministerial Order, 140, had as a significant motivator the publication of Law 12.732 of November 22, 2012, which established a maximum deadline of 60 days for the start of treatment of patients diagnosed with cancer 20.

The Portaria changed the focus of habilitation, leading to the need for regional discussions and the elaboration of care plans that considered the network that included the establishment to be enabled. It was no longer sufficient to forward the request of the qualifying hospital describing its structure and capabilities to perform this highly complex care. The managers needed to describe how organization and responsibility would be allotted at all levels of care, considering all of the components of the network of care for people with chronic diseases in the thematic axis of cancer [i.e., Primary Care, Home Care, Ambulatory Specialised Care, Specialised Hospital Care (Centro de Alta Complexidade em Oncologia; CACON; Unidade de Assistência de Alta Complexidade em Oncologia; UNACON), Complexes - General Hospital with cancer surgery in the Hospital Complex, Radiotherapy Service of Hospital Complex, Support Systems, Regulation, Logistical Systems and Governance].

Concern about establishing a network of cancer care in the State was emphasized in 1991 with the creation of ONCO-REDE (State Network of Tertiary Oncology Assistance). In a Decree of the Governor, the creation of a network within the scope of the State Department of Health composed of the CECANs (Cancer Centres), or public and private hospitals belonging to the SUS, was defined as providing assistance, educational and scientific activities in oncology by the Oncology Foundation of São Paulo (Fundação de Oncocentro de São Paulo; FOSP) and by public and private entities in support of cancer patients 21. A study of 178,570 cases from the Hospital Registry of Cancer between 2000 and 2004 showed a gradual increase in morbidity in both females and males, with almost half of the cases (47.8%) requiring services for advanced stages of the disease (stages III and IV). The number of oncological surgeries, chemotherapy and radiotherapy procedures increased to 0.5% and 9.1% of expenditures, with a 111% increase in expenses 22.

In this context, on May 8, 2008, the activities of the Cancer Institute of the State of São Paulo (ICESP) began in the management model of the Social Health Organisations (Organização Social de Saúde; OSS), with technical and scientific coordination of the Faculty of Medicine of the University (Faculdade de Medicina da Universidade de São Paulo; FMUSP). In the OSS management model, a private organization administers the hospital through a contract that provides goals and results. These activities were structured to provide comprehensive care to adult cancer patients at all stages of treatment and for all of their needs, including palliative care when necessary 23. Installed in a 28-story building were 499 beds for hospitalization, 11 operating rooms, 100 armchairs for outpatient chemotherapy infusion, 63 surgeries and 6 linear accelerators. An outpatient care facility, a rehabilitation center, and an outpatient pharmacy for patients under treatment as well as a referral center for palliative care were installed in an out-of-the-way facility 50 kilometers away. The workforce consisted of 3,632 employees, of whom 569 were physicians and 704 were service providers from third-party companies in the areas of concierge, security, reception, nutrition, hygiene and cleaning, IT and building maintenance. Patients with a diagnosis of cancer were admitted to the ICESP via referral from one of the diagnostic units (AMEs; Ambulatorio Médico de Especialidades) and state and/or municipal hospitals in São Paulo and the metropolitan region, a process coordinated by a Regulation Center of Patients using SUS principles and technical referral criteria. An average of 1,000 new patients started treatment each month, triggering a monthly average of 4,559 chemotherapy sessions, 4,950 radiotherapy sessions, 608 surgeries and 25,058 outpatient clinic visits to the multiprofessional team 24.

In almost three years of operation, ICESP has established itself as the technical reference in oncology for the State Department of Health (Secretaria Estadual de Saúde; SES). It is the only SES hospital dedicated to the treatment of adult patients with cancer (all types) in the SUS, with two years of activity. It published the Manual of Pipes in Oncology, which provided protocols of conduct specific to each type of cancer, based on extensive discussion with the specialists of the Department of Radiology and Oncology of FMUSP and members of the clinical staff of ICESP. The criteria for choosing the best conduct involved a broad review of the literature and, pragmatically, behaviors with evident efficacy and possibilities of use in the SUS context 25.

In the State Health Plan for the period from 2008 to 2011, oncology appears with a focus on the organization of the oncology network in the health regions and in the formation of multiprofessional teams for the three levels of organization and the goal of reducing mortality. In the elaboration of this plan, the need to expand the service offer was identified in the 64 units qualified for oncology care in the SUS. This service was offered from July 2005 to July 2006, and 70% of the oncological surgeries and 85% of the chemotherapies were required, as were 43% of the radiotherapies 26. 

For the following period (State Health Plan 2012 to 2015), cancer mortality was ranked second with a growing trend, accounting for 15.6% of deaths. The State had 71 units qualified for oncological care in the SUS. There were concerns about the reorganization of the Oncology Network that was already installed and the integration of the services into Regional Health Care Networks (RRAS) 27. For this purpose, a Situational Diagnosis of Oncological Care in the State was elaborated, demonstrating an unequal distribution of the qualified units in the RRAS with no coherence between the population and available services. We identified a deficit in the provision of radiotherapy services, insufficiency in oncological surgeries, and a concentration of chemotherapy and radiotherapy production in 10 institutions. In addition, a challenge related to early detection was observed since 50.1% of the patients who arrived at the services for treatment were in clinical stages II, III and IV 28.

Based on this situation, a Cancer Care Plan for the State of São Paulo was proposed for the period of 2011 to 2014 with the objective of reducing cancer incidence and mortality, increasing survival and improving the quality of life of patients (Table 2). The Advisory Committee on Oncology of the State of São Paulo as established for technical implementation and was directly linked to the Office of the State Department of Health. It consisted of representatives of 13 CACONs from FOSP and was coordinated by a representative of ICESP. To assist in the work of the Committee, an Executive Secretariat was established consisting of technicians from the State Department of Health (Coordination of Health Regions, Planning Coordination, Health Services Coordination and Health Service Contracts Management Coordination), FOSP, COSEMS representatives and ICESP technicians under the coordination of the latter. For the development of this work, the Executive Office of the Oncology Network was structured within ICESP as an advisory to the Executive Board of the ICESP. With a routine of ordinary monthly meetings, the members of the Committee began to investigate the demands sent by the State Department of Health and to send recommendations via technical opinions to have their dynamics and organization defined in the Internal Rules.

## DISCUSSION

Examining the model proposed by Shortell, this work attempted to evaluate the “new culture of management” through the analysis of the rules and regulations produced by the Ministry of Health for Oncology. As an area classified within the High Complexity of SUS, in which the manager requires compliance with minimum parameters for the service provider, Ministerial Ordinances are important drivers of the establishment of care models and the implementation of management culture. It is possible to observe a direction for integral and integrated care. It is necessary to evaluate the practices adopted from these criteria and determine whether they were adopted to complete this analysis. Since the Ministry of Health published the first ordinance with criteria for accreditation and habilitation of treatment services to cancer patients, more than 20 years have passed. During this period, it is possible to observe a progressive orientation towards the integral and integrated care of patients with cancer. In 2005, with the definition of the oncology care line of the National Policy on Cancer Care, which was an important component of the establishment of a clinic management culture, the establishment of a deadline for the end of isolated chemotherapy and radiotherapy services in the Portaria SAS No. 741 was identified. In 2014, the requirement of the referral of a Regional Plan and the candidate services for qualification in oncology, together with the local managers, demonstrated the ability to establish a network of patient care at the three levels of organization (for example, using management tools such as regulation). To do this, the hospital can offer the structure and services needed for every step of the care line, focus on care that requires more expensive and complex technologies and establish referral and reference flows with other levels of care.

In this context, the State Department of Health of São Paulo adopted a strategy of inviting representatives of this specialty, oncology representatives from hospitals qualified in the SUS with high production, and established a governance system with technical-scientific forums and executive support to SES with the participation of the FOSP and with a main base in ICESP. ICESP has become the reference for the State due to its management model and its connection with the Faculty of Medicine of the University of São Paulo. The State assumed the leading role in the organization of a thematic network, and the experts were able to contribute to the definition of public policies. This model reproduced the national model of INCA as a technical-scientific reference for the Ministry of Health.

Although we have observed evolution in the regulation of cancer care in SUS, there are still many challenges for the establishment of the Oncology Network in the integrated care model. The coexistence of the specialists of the Reference Committee on Oncology with the technicians of the Health Department, the managers of the system, showed on the one hand the need for this approach to establish technical parameters to support the decisions and organization of services. On the other hand, there is still difficulty, from all elements of the system, in prioritizing the adoption of management tools for clinical practice and regulation. The examples of projects presented in this article reinforce the idea that specialized hospitals should participate more actively in the discussions and elaboration of public health policies and, in this way, may participate in health care networks in a role that goes beyond the care of acute chronic conditions, contributing to the development of clinical management technologies and alternative processes.

## AUTHOR CONTRIBUTIONS

Written by Madi MR with guidance from Cerri GG.

## Figures and Tables

**Table 1:** Ministry rules related to cancer care in the SUS - 1993 to 2014.

Year	Ministries rules	Objective	Implementation
1993	Portaria SAS/MS n° 65	Recognizes oncology as a highly complex network: Integrated Networks of Procedures of High Complexity, Cardiology, Oncology, Orthopaedics, Ophthalmology and Organ Transplants	State Health Departments indicated the services that should be accredited and/or disqualified in addition to the monitoring and evaluation of the procedures
1993	Portaria SAS/MS n° 170	Accreditation standards: Reference Center I and II for hospitals, with I being totally dedicated to oncology and more complete; radiotherapy and chemotherapy centers isolated from hospitals	Executive Secretary of High Complexity in Cancer at INCA, together with the Advisory Board of INCA, made a technical evaluation in the accreditation processes of oncology centers throughout the country
1998	Portaria GM/MS n° 3535	Mandatory minimum services for all CACONs (I, II and III): prevention, early detection, diagnosis and treatment of patients in seven modalities: diagnosis, oncological surgery, clinical oncology, radiotherapy, support measures, rehabilitation and palliative care	INCA continues as a technical advisory body of the Ministry of Health to advise on the accreditation of services. It established parameters for the sizing of services according to the number of estimated new cases and INCA
2005	Portaria GM/MS n° 2439	National Cancer Care Policy: Unit of High Complexity in Oncology (UNACON) and CACON (Center of Reference in High Complexity of Oncology)	Definition of the care line and technical criteria for the operation and evaluation of public and private services that work at different levels of cancer care; definition of a CACON regional reference
2005	Portaria SAS/MS n° 741	New accreditation rules for all services: Isolated chemotherapy and radiotherapy services that could continue to function under specific conditions but linked to a UNACON or CACON with predicted technical cooperation and joint planning of treatments	Definition of parameters for more detailed planning and evaluation of the network and based on the INCA estimates of number of new cases per region; minimum number of production per treatment modality
2014	Portaria SAS/MS n° 140	CACON or UNACON Hospital Complexes Radiotherapy Service of Hospital Complex and/or General Hospitals with Oncologic Surgery of Hospital Complex	Changed the focus of habilitation, bringing the need for regional discussions and elaboration of care plans that consider the network where the establishment to be enabled is inserted - Regional Cancer Attention Plan

Source: Brasil, Ministério da Saúde - 1993 to 2014.

**Table 2:** Guidelines and perspectives of the Oncology Care Plan for the State of São Paulo - 2011 to 2014.

Cancer Care Plan	
**Perspective: promotion and protection of health**	
Perspective 1	Strengthening of healthy lifestyle promotion actions
Perspective 2	Specific health education program for children and adolescents
Perspective 3	Increased actions to support smoking cessation
Perspective 4	Education for the prevention of cancer in different media
Perspective 5	Information and awareness for target groups for cancer screening
**Perspective: early detection**	
Perspective 6	Policies for screening for cervical, breast and colorectal cancer
Perspective 7	Shift from opportunistic to population-based tracking model
Perspective 8	Planning and design to implement colorectal cancer screening
**Perspective: patient care**	
Perspective 9	Tools for quantifying and qualifying the care network
Perspective 10	Elaboration of protocols for diagnosis, treatment and follow-up
Perspective 11	Implement regulation of access to care
Perspective 12	Implement the State Oncology Network
Perspective 13	Actions to scale the accredited network and identify regional needs
Perspective 14	Plan to expand the radiotherapy park
Perspective 15	Establish mechanisms to evaluate the accredited network
**Perspective: palliative care and pain**	
Perspective 16	Define regional needs for palliative care in an articulated way with other health areas
Perspective 17	Implement studies to define models of palliative care
Perspective 18	Develop studies and actions for pain relief policy in oncology

Source: Correa et al., 2012.
